# Supplementary material for: A Community-Based Participatory Framework to Co-Develop Patient Education Materials (PEMs) for Rare Diseases: A Model Transferable across Diseases
Source: Int J Environ Res Public Health. 2023 Jan 5;20(2):968. doi: 10.3390/ijerph20020968 (PMC9859511; doi:10.3390/ijerph20020968)
Supplement: Supplementary file 1 [file ijerph-20-00968-s001.zip › ijerph-2085282-supplementary.pdf]

Supplementary materials

Table S1 Health Literacy definitions evolution. Since its first use at a health education conference in 1974, the definition of health literacy has evolved as Frisch and colleagues point out [2,3]. After 1992 the use of health literacy has increased and the importance of health literacy for public health and healthcare is growing. The majority of research literature on health literacy has been published in 2005 onwards [4]. The wide range of health literacy definitions, the continued discussions on whether health literacy is an individual or system skill, if it is stagnant or dynamic, all contribute to the challenges of continued research, consistent measurement, and possible solutions to enhancing low health literacy.

Author(s)	Year	Definition
World Health Organization[5]	1998	"Cognitive and social skills which determine the motivation and ability of individuals to gain access to, understand, and use information in ways that promote and maintain good health."
American Medical Association[6]	1999	"The constellation of skills, including the ability to perform basic reading and numerical tasks required to function in the health care environment."
Nutbeam[7]	2000	"The personal, cognitive, and social skills which determine the ability of individuals to gain access and understand, and use information to promote and maintain good health."

Institute of Medicine[8]	2004	"The individuals' capacity to obtain, process and understand basic health information and services needed to make appropriate health decisions"
Kickbusch, Wait & Maag[9]	(2005)	"The ability to make sound health decision(s) in the context of everyday life--at home, in the community, at the workplace, the healthcare system, the market place and the political arena. It is a critical empowerment strategy to increase people's control over their health, their ability to seek out information and their ability to take responsibility"
Zarcadoolas, Pleasant, & Greer [10-12]	2003, 2005, 2006	“A wide range of skills that people develop to seek out, comprehend, evaluate and use health information and concepts to make informed choices, reduce risks and increase quality of life.”
McCabe[13]	2006	“A tapestry of skills combining basic literacy, math skills, and a belief in the basic tenets of the treatment modality.”

Paasche-Orlow & Wolf [14]	2006	"An individual's possession of requisite skills for making health-related decisions, which means that health literacy must always be examined in the context of the specific tasks that need to be accomplished. The importance of a contextual appreciation of health literacy must be underscored"
EU[15]	2007	"The ability to read, filter and understand health information in order to form sound judgments"
Pavlekovic[16]	2008	"The capacity to obtain, interpret and understand basic health information and services and the competence to use such information to enhance health"
Rootman & Gordon-Elbihbety[17]	2008	"The ability to access, understand, evaluate and communicate information as a way to promote, maintain and improve health in a variety of settings across the life course"

Ishikawa & Yano[18]	2008	"The knowledge, skills and abilities that pertain to interactions with the healthcare system"
Mancuso[19]	2008	"A process that evolves over one's lifetime and encompasses the attributes of capacity, comprehension, and communication. The attributes of health literacy are integrated within and preceded by the skills, strategies, and abilities embedded within the competencies needed to attain health literacy"
Australian Bureau of Statistics[20]	2008	"The knowledge and skills required to understand and use information relating to health issues such as drugs and alcohol, disease prevention and treatment, safety and accident prevention, first aid, emergencies, and staying healthy"

Yost et al.[21]	2009	"The degree to which individuals have the capacity to read and comprehend health-related print material, identify and interpret information presented in graphical format (charts, graphs and tables), and perform arithmetic operations in order to make appropriate health and care decisions"
Adams et al.[22]	2009	"The ability to understand and interpret the meaning of health information in written, spoken or digital form and how this motivates people to embrace or disregard actions relating to health"
Adkins et al.[23]	2009	"The ability to derive meaning from different forms of communication by using a variety of skills to accomplish health-related objectives"
Freedman et al.[24]	2009	“The degree to which individuals and groups can obtain, process, understand, evaluate, and act upon information needed to make

		public health decisions that benefit the community."
Berkman, Davis, & McCormack[25]	2010	"Dependent upon individual and system factors, which also include the communication skills, knowledge, and culture of both the professional and lay person, the context as well as the demands of the health care and public health system."
Patient Protection and Affordable Care Act of 2010[26]	2010	"The degree to which an individual has the capacity to obtain, communicate, process, and understand basic health information and services to make appropriate health decisions."

Sorensen et al.[27]	2012	"Health literacy is linked to literacy and entails people's knowledge, motivation, and competence to access, understand, appraise and apply health information in order to make judgements and take decisions in everyday life concerning healthcare, disease prevention, and health promotion to maintain or improve quality of life during the life-course."
Healthy People 2010 and Healthy People 2020[28]	2010 e 2020	"the degree to which individuals have the capacity to obtain, process, and understand basic health information and services needed to make appropriate health decisions."

Healthy People 2030[28]	Until 2030	“Personal health literacy is the degree to which individuals have the ability to find, understand, and use information and services to inform health-related decisions and actions for themselves and others.<br><br>Organizational health literacy is the degree to which organizations equitably enable individuals to find, understand, and use information and services to inform health-related decisions and actions for themselves and others.

2. Frisch AL, Camerini L, Diviani N, Schulz PJ. Defining and measuring health literacy: How can we profit from other literacy domains? Health Promot Int. 2012. p. 117–26.

3. Robinson-Pant A 1960-. Promoting health and literacy for women’s empowerment. Hamburg: UNESCO Institute for Lifelong Learning; 2016.

4. Speros C, Aprn D. Health literacy: concept analysis. J Adv Nurs. 2005;50:633–40.

5. Nutbeam D. Health promotion glossary. Health Promot Int. 1998;13:349–64.

6. Bresolin LB. Health literacy: Report of the council on scientific affairs. JAMA. American Medical Association; 1999;281:552–7.

7. Nutbeam D. Health Literacy as a public health goal: a challenge for contemporary health education and communication strategies into the 21st century. Health Promot Int. 2006;15:259–67.

8. Institute of Medicine (US): Committee on Health Literacy. Health Literacy: A Prescription to End Confusion. Nielsen-Bohlman L, Panzer AM, Kindig DA, editors. Washington, D.C.: National Academies Press; 2004.

9. Kickbusch I, Wait S, Maag D. Navigating Health: The Role of Health Literacy. 2006.

10. Zarcadoolas C, Pleasant A, Greer DS. Elaborating a definition of health literacy: A commentary. J Health Commun. 2003. p. 119–20.

11. Zarcadoolas C, Pleasant A, Greer DS. Understanding health literacy: An expanded model. Health Promot Int. 2005. p. 195–203.

12. Zarcadoolas C, Pleasant A, Greer D. Advancing Health Literacy: A Framework for Understanding and Action. 2006.

13. McCabe JA. An assignment for building an awareness of the intersection of health literacy and cultural competence skills. J Med Libr Assoc. 2006;94:458–61.

14. Paasche-Orlow MK, Wolf MS. The causal pathways linking health literacy to health outcomes. Am J Health Behav. PNG Publications; 2007;31.

15. European Commission. Together for Health-A Strategic Approach for the EU 2008-2013. 2007; Available online: http://ec.europa.eu/health/ph_overview/strategy/health_strategy_en.html (accessed 22 November 2022)

16. Pavlekovic G. Health Literacy. Programmes for Training on Research in Public Health for South Eastern Europe. Vol. 4 2008.

17. Rootman Irving, Gordon-El-Bihbety Deborah, Canadian Public Health Association. Expert Panel on Health Literacy., Gibson Library Connections. A vision for a health literate Canada : report of the Expert Panel on Health Literacy. Canadian Public Health Association; 2008.

18. Ishikawa H, Yano E. Patient health literacy and participation in the health-care process. Health Expectations. 2008. p. 113–22.

19. Mancuso JM. Health literacy: A concept/dimensional analysis. Nurs Health Sci. 2008;10:248–55.

20. Australian Bureau of Statistics. Adult Literacy and Life Skills Survey, Summary Results. 2006.

21. Yost KJ, Webster K, Baker DW, Choi SW, Bode RK, Hahn EA. Bilingual health literacy assessment using the Talking Touchscreen/la Pantalla Parlanchina: Development and pilot testing. Patient Educ Couns. 2009;75:295–301.

22. Adams RJ, Stocks NP, Wilson DH, Hill CL, Gravier S, Kickbusch I, et al. Health literacy--a new concept for general practice? Aust Fam Physician. 2009;38:144–7.

23. Adkins N, Corus C. Health Literacy for Improved Health Outcomes: Effective Capital in the Marketplace. J Consum Aff. 2009;43:199–222.

24. Freedman DA, Bess KD, Tucker HA, Boyd DL, Tuchman AM, Wallston KA. Public Health Literacy Defined. Am J Prev Med. 2009. p. 446–51.

25. Berkman ND, Davis TC, McCormack L. Health literacy: What is it? J Health Commun. 2010. p. 9–19.

26. Rosenbaum S. The Patient Protection and Affordable Care Act: Implications for Public Health Policy and Practice. 2011.

27. Sørensen K, van den Broucke S, Fullam J, Doyle G, Pelikan J, Slonska Z, et al. Health literacy and public health: A systematic review and integration of definitions and models. BMC Public Health. 2012.

28. Office of the Assistant Secretary for Health. Available online: https://health.gov/healthypeople/priority-areas/health-literacy-healthy-people-2030 (accessed 22 November 2022)

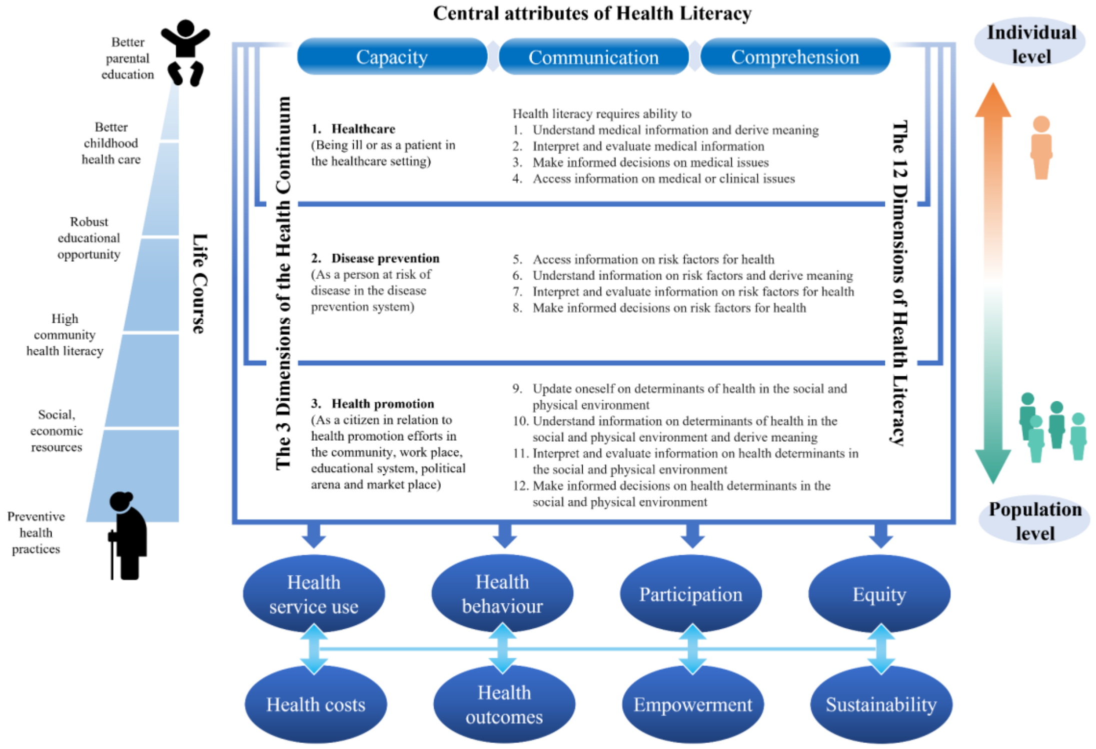

Figure S1 Combination of four competences referring to health information processing with the three levels of domains named healthcare, disease prevention and health promotion yields a matrix of 12 subdimensions of health literacy.

Table S2 Keywords list to search through the Medline database using PubMed as the search engine with a double and triple combination of terms related to rare diseases, CDG, and PEMs.

**Keyword 1 combined with**	**Keyword 2**	**Keyword 3**
Health literacy	Rare diseases	
Health literacy	Plain language	Rare disease
Plain language	Rare disease	
Patient education materials	Plain language	Patient empowerment
Patient education materials	Plain language	Rare disease
Rare disease	Plain language	Patient empowerment
Patient education materials	Plain language	
Patient education materials	Patient engagement	

Patient education materials	Patient engagement	Rare disease
Health literacy	Patient empowerment	
Health literacy	Patient empowerment	Rare disease
Patient education materials	Community based participatory research	
Patient education materials	Community based participatory research	Rare disease
	Community based participatory research	Rare disease
Patient education materials	Congenital Disorders of Glycosylation (CDG)	
Patient education materials	Congenital Disorders of Glycosylation (CDG)	Patient empowerment
	Congenital Disorders of Glycosylation (CDG)	Patient empowerment
Health literacy	Patient empowerment	Congenital Disorders of Glycosylation (CDG)
Patient education materials	Rare diseases	
Patient education materials	Rare diseases	Patient empowerment
Patient education materials	People centricity	
Health literacy	People centricity	Patient empowerment
Readability score	Patient education materials	
Health literacy	Readability score	
Shared decision making	Patient education materials	Rare diseases
Health education	Shared decision making	
Health education	Shared decision making	Rare disease

Table S3 CDG and Allies - PPAIN and the Portuguese Association for CDG (APCDG), different types of educational materials

**Educational materials available at World CDG Org**	**Topic**	**Brief Description**	**Direct Link To the material**
CDG community tailored content and materials	What is CDG	Information available on WorldCDG.org	(https://worldcdg.org/about-cdg/what-cdg)
Community friendly guide	Practical guide CDG	Document with guidelines on CDG	
Glossaries	Immunological involvement in Congenital Disorders of Glycosylation (CDG)	Several mini-glossaries, each dedicated to a specific topic related with CDG and immunology, including: Immune system, Immunological tests, Nutritional supplementation for CDG, Types of infections, Agents of infection, autoimmune disease and allergy	
	Prioritizing Symptoms Impacting Quality of Life for CDG	Several glossaries have been prepared in order to walk participants throughout:<br>• CDG Signs & Symptoms	https://worldcdg.org/research-cdg-journey-mapping/survey-1-prioritizing-symptoms-impacting-quality-life-cdg

		● Treatments<br>● Diets<br>● Management & Rehabilitation Therapies<br>● Health Care Specialties	
Infographics	Total 23	Summary on CDG in Lay Language	https://worldcdg.org/resources?resources=&type=266
Summaries	Total 30	Summary on CDG in Lay Language	Under email request
Posters	ImmunoCDGQ: Immunology and CDG Questionnaire for Patients and Caregivers	Document in Poster format	
Scientific Papers	The road to successful people-centric research in rare diseases: the web-based case study of the Immunology and Congenital Disorders of Glycosylation questionnaire (ImmunoCDGQ)	Manuscript	https://pubmed.ncbi.nlm.nih.gov/35331276/)
Oral communications	Novel insights into immunological involvement in Congenital Disorders of Glycosylation (CDG) through a people-centric approach	Powerpoint apresentation and videos	

Guidelines	PMM2-CDG: International Clinical Guidelines for the Management of Phosphomannomutase 2-congenital Disorders of Glycosylation: Diagnosis, Treatment and Follow Up	Document with the guidelines for PMM2-CDG	
Webinar	CDG Virtual Coffee	Video	https://www.youtube.com/watch?v=ZWzUnzSspDc&list=PLHxBb6YfsCdMc-Tl5EwQaqoIoQB4PVCIw
Children's book	Glicolandia y las antenas de colores (castellano).	Document made for children to teach them about CDG	
Interview	Adaptive parenting strategies in CDG families	Video with the testemony of CDG Families	
Social media Campaign	CDG: A community of Hope (https://www.youtube.com)	Videos and social media publications	https://www.youtube.com/watch?v=S1D_yyNdZuc&list=PLHxBb6YfsCdPMnFQmmrgP4d6mD70ig4xv
Magazine spotlight	Rare revolution magazine digital spotlight: A carer's perspective: the impact a diagnosis of congenital disorders of glycosylation (CDG) has on a family	Interviews in document format	

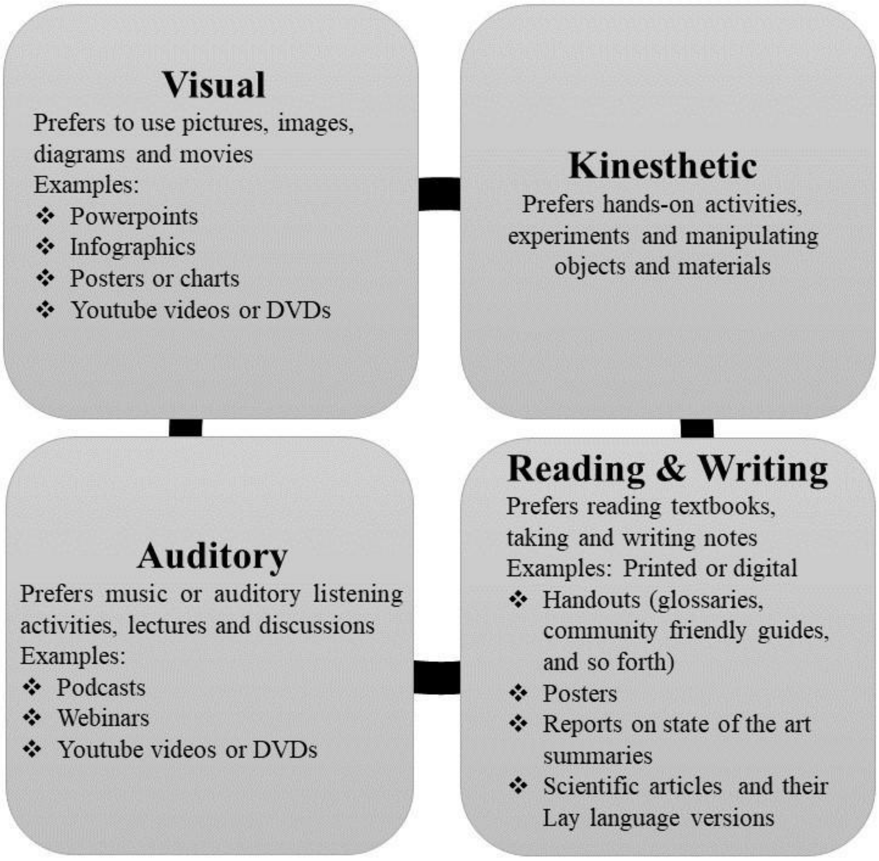

Figure S2 - Overview of Patient Education Materials: Each audience has a learning styles, and once it is identified, several best format for patient education materials are available: VARK Learning Styles (Cuevas, 2015: https://journals.sagepub.com/doi/full/10.1177/1477878515606621)

Table S4 - Keywords list to search through the Medline database using PubMed as the search engine with a double and triple combination of terms related to rare diseases, Internet platforms and trnslate scientific language

**Keywords**		
Column 1 and Column 2		and then also combine with Column 3
"internet platform" simplify	"biomedical language"<br>"medical language"<br>"scientific language"	rare diseases
"internet platform" summarize		
"internet platform" translate		
"App summarize"		
App simplify translate		
"AI-based software platform simplify"		
"AI-based software platform summarize"		

“AI-based software platform translate ”		
“Simplify”		
“Summarize”		
“Digital health platform”	“Biomedical”<br><br>“Science”<br><br>“Medical”	
"Lay language"		
“Deep learnings language processing to simplify”	“Biomedicine”<br><br>“Research”<br><br>“Science”	
“Deep learnings language processing to summarize”		
“Deep learnings language processing to translate		
“Artificial neural network language processing to simplify”		

“Artificial neural network language processing to summarize”		
“Artificial neural network language processing to translate”		
“AI language processing to simplify”		
“AI language processing to summarize”		
“AI language processing to translate”		
“machine learning language processing to simplify”		
“machine learning language processing to summarize”		
“machine learning language processing to translate”		

“AI-Based Tool To Summarize”	“Biomedical Papers”<br>“Research Papers”<br>“Medical Papers”<br>“Scientific Papers”	
“Machine learning-Based Tool To Summarize”		
“Artificial neural network-Based Tool To Summarize”		
“Internet platform simplify”	“Biomedical jargon”<br>“Medical Jargon”<br>“Scientific Jargon”	
“Internet platform summarize”		
“Internet platform translate”		
“Text Simplification” for	“Scientific Information Access”	

	“Medical Information Access”	
“Internet platform simplify”	“Biomedical vocabulary”<br>“Scientific vocabulary”<br>“Medical vocabulary”*	
“Internet platform summarize”		
“Internet platform translate		
“platform simplify”	“scientific language”<br>“Medical language”	
“platform summarize”		
“platform translate”		
“digital platform[2] ”	"lay language" health	
“open- source AI trained on open data depositories[3] “	“reviews documents”<br>“journal documents”<br>“medical documents”	

“Automated Lay Language Summarization[4] ”	“Biomedical Reviews”<br><br>“Medical Reviews”<br><br>“Scientific Reviews”<br><br>“Biomedical articles”<br><br>“Medical articles”<br><br>“Scientific articles”<br><br>“Biomedical journals”<br><br>“Medical journals”<br><br>“Scientific journals”	

digital platform "lay language	“rare diseases”		
“Scientific simplification” [5]			
“Medical text simplification [6] “			
(Multi-document) scientific			
“medical summarization ”			
“Deep learnings”			

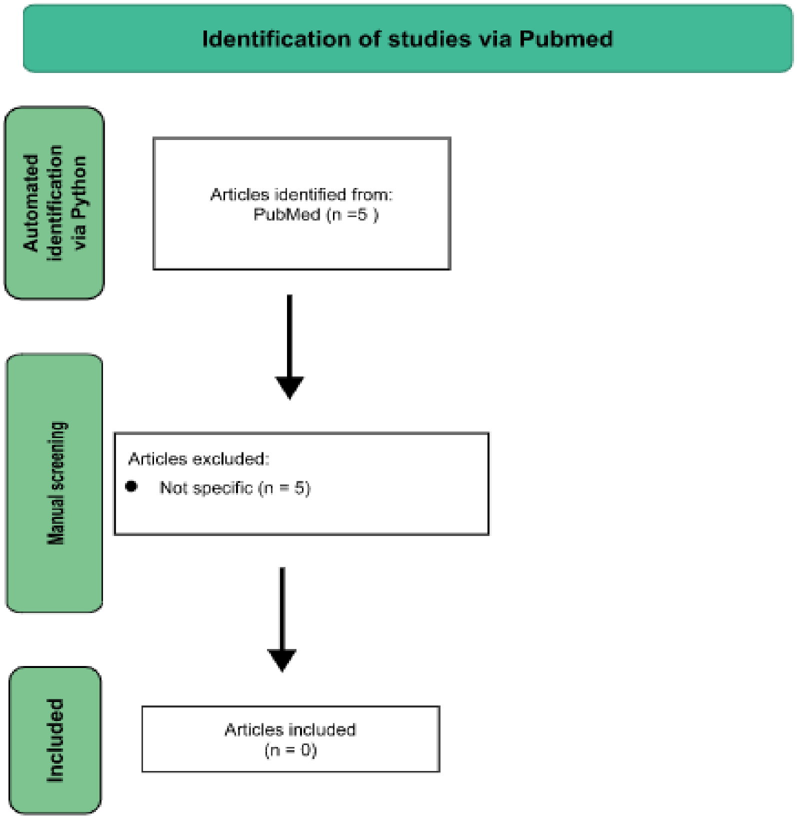

Figure S3 - Flow diagram for the systematic review which included searches of databases. Only articles related toInternet platforms that translate scientific language developed for rare diseases were included. The PRISMA flow diagram was adapted from the study by Page et al (Page, M.J.; McKenzie, J.E.; Bossuyt, P.M.; Boutron, I.; Hoffmann, T.C.; Mulrow, C.D.; Shamseer, L.; Tetzlaff, J.M.; Akl, E.A.; Brennan, S.E.; et al. The PRISMA 2020 statement: An updated guideline for reporting systematic reviews. PLoS Med. 2021, 18,e1003583.)
